# Supplementary material for: Construction of a Ginseng Root-Meristem Sensor and a Sensing Kinetics Study on the Main Nitrogen Nutrients
Source: Sensors (Basel). 2021 Jan 20;21(3):681. doi: 10.3390/s21030681 (PMC7863967; doi:10.3390/s21030681)
Supplement: Supplementary file 1 [file sensors-21-00681-s001.pdf]

SUPPLEMENTARY DATA

Construction of a Ginseng Root-Meristem Sensor and a Sensing Kinetics Study on the Main Nitrogen Nutrients

Shiang Wang, [a,b] Dingqiang Lu, [a,b*] Guangchang Pang [a, b*]

[a] College of Biotechnology & food Science, Tianjin University of Commerce, Tianjin 300314, China

[b] Tianjin key laboratory of food biotechnology, Tianjin 300314, China

[*] Corresponding author: Tel: +86-2226675780; fax: +86-2226675780

E-mail address: dqlu@tjcu.edu.cn (D.Q.Lu); pgc@tjcu.edu.cn (G.C.Pang)

Fig.S1

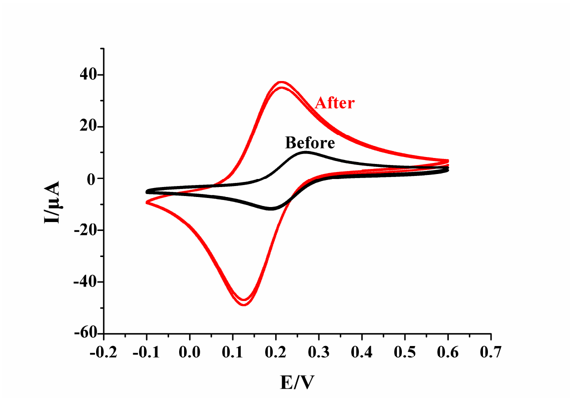

Fig. S1 Cyclic voltammetry characterization before and after the pre-treatment of bare electrode. As shown in Figure S1, the redox peak current increased considerably, the peak current ratio was close to 1, and the peak potential difference was less than 80 mV.

Fig.S2

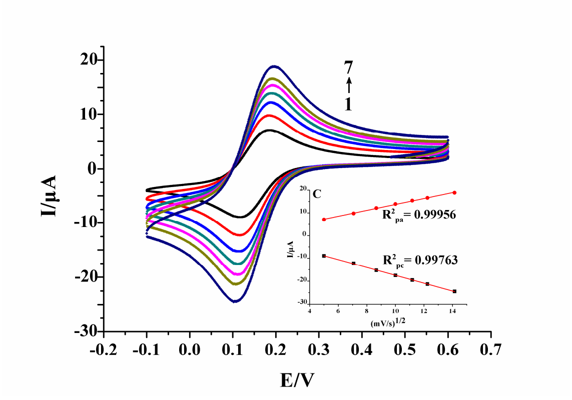

Fig. S2 Cyclic voltammograms of the bare electrode at different scan rates (0.025 V/s, 0.050 V/s, 0.075 V/s, 0.100 V/s, 0.125 V/s, 0.150 V/s, and 0.200 V/s). The inset shows that the redox peak current had a good linear relationship with the square root of the scan rate, indicating that the redox peak current was only controlled by diffusion.

Fig.S3

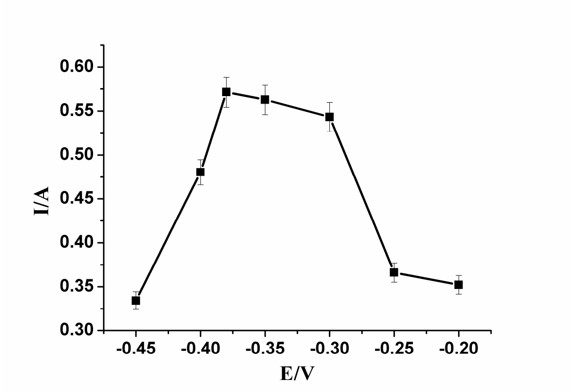

Fig. S3 Optimization of detection potential. 1 × 10^{-20} mol/L sodium nitrate solution [blank solution] was used as an example in the figure. The change in the steady-state current value before and after the addition of five types of nitrogen source solutions was used to measure the influence of electric potential on the electrochemical performance of the plant sensor. As shown in Figure S3, the current value was the largest when the voltage was −0.38 V, and the current change was the largest when the voltage was less than −0.38 V. The above results demonstrated, first, that the sensing signal was the strongest when a constant potential of −0.38 V was used during the measurement of the five nitrogen source solutions with the ginseng root meristem sensor; second, that the sensing of the five nitrogen sources by the root meristem could be effectively realized; and third, that noise interference could be avoided.

Fig.S4

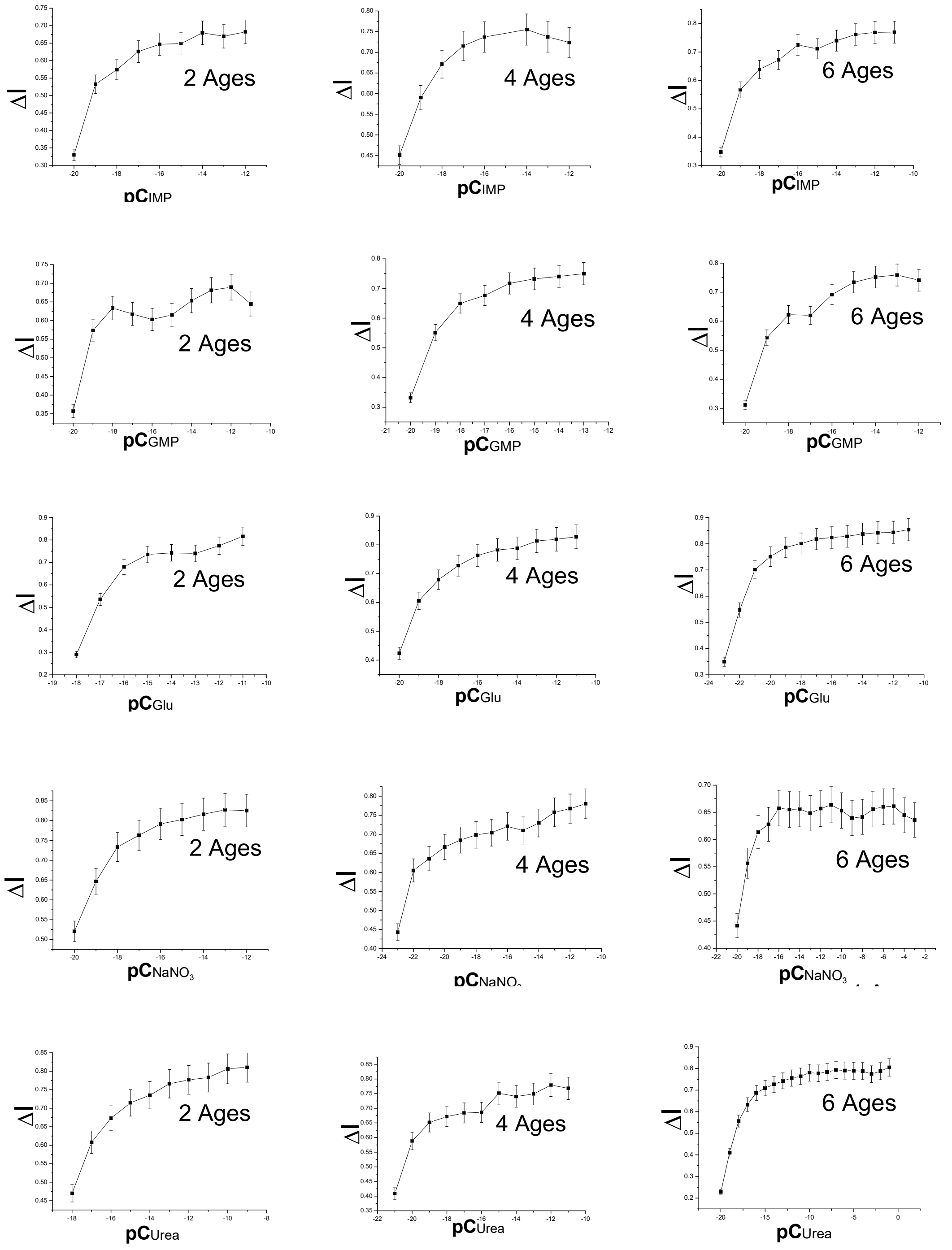

Fig. S4 Rate of current change of five nitrogen nutrients in the detection concentration range of two-, four-, and six-year-old ginseng root-meristem sensor. The results show that the rate of response current was positively correlated with concentration.

Fig.S5

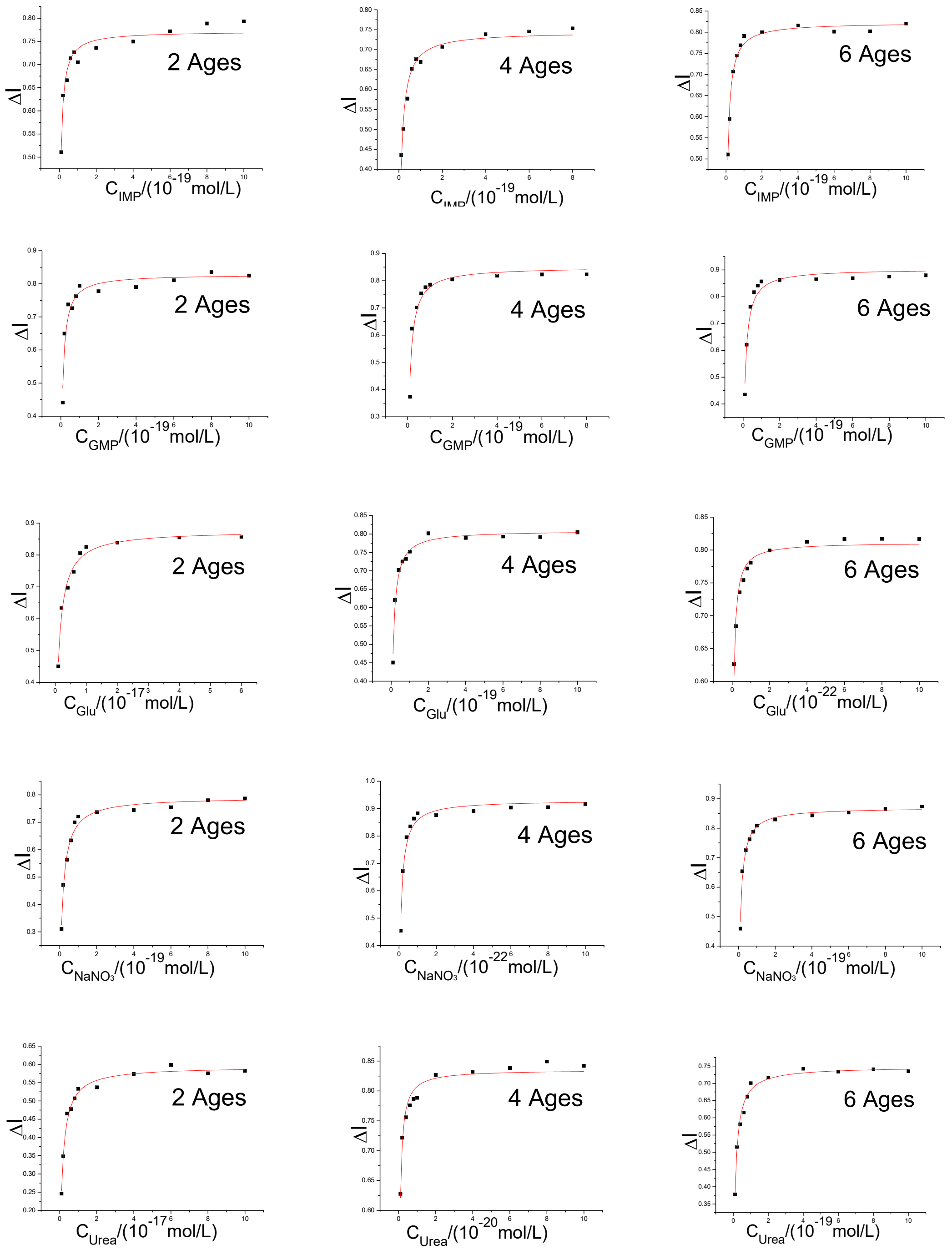

Fig. S5 Response curve fitting of two-, four-, and six-year-old ginseng root-meristem sensor to five nitrogen nutrients in the concentration range.

Fig.S6

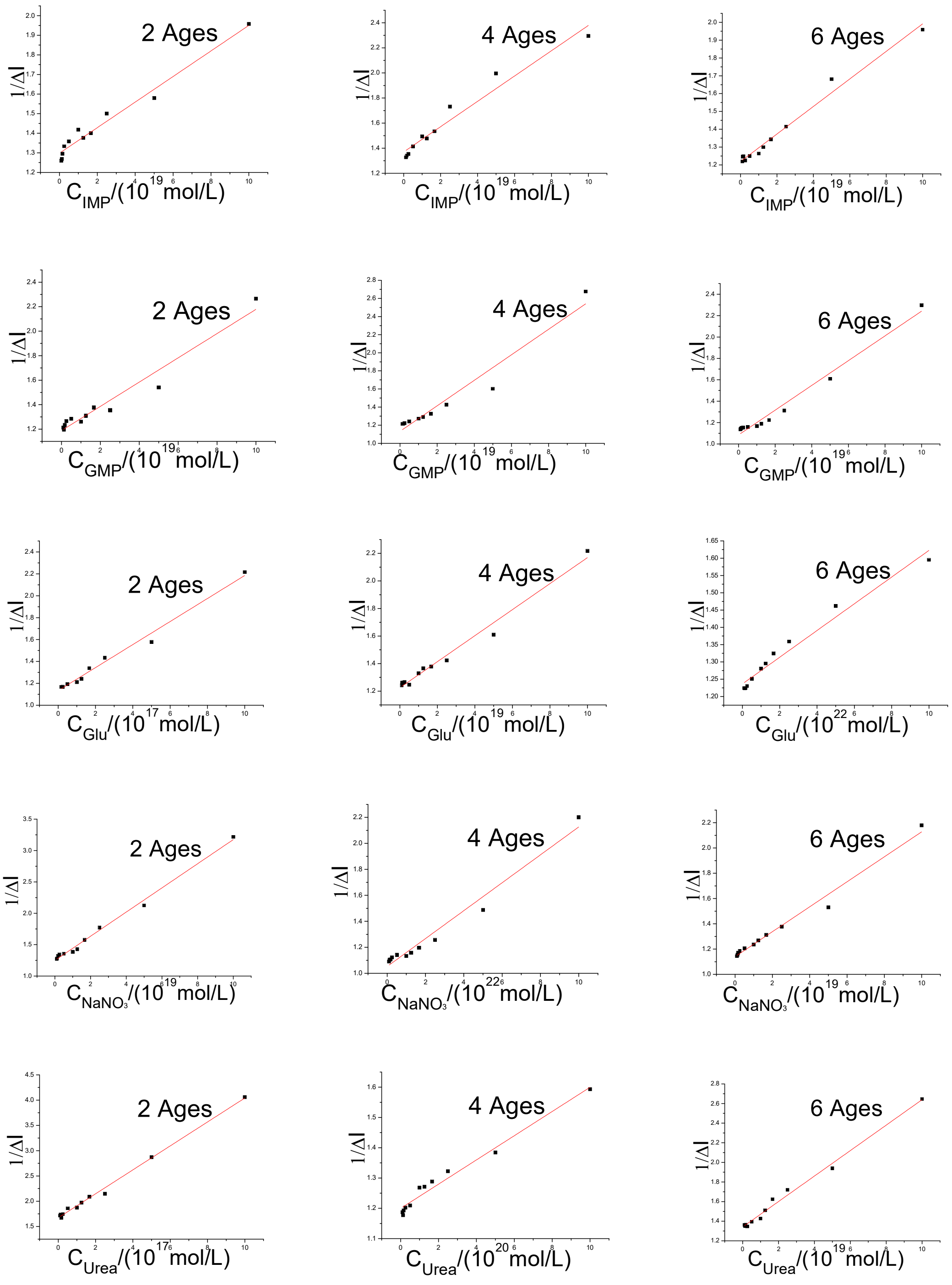

Fig. S6 Double reciprocal pseudo-curve of five nitrogen nutrients in the concentration range of two-, four-, and six-year-old ginseng root-meristem sensor.
